# The predictive role of coach–athlete relationship quality in training engagement and skill development among adolescent basketball players

**DOI:** 10.3389/fpsyg.2025.1648082

**Published:** 2025-08-29

**Authors:** Yuanyuan Luo, Shuairan Li, Yingying Cao, Zhongjian Luo

**Affiliations:** 1 Dazhou Vocational College of Chinese Medicine, Dazhou, China; 2 Sports Coaching College, Beijing Sport University, Beijing, China; 3 School of Sports, Xi'an University, Xi'an, China

**Keywords:** coach–athlete relationship, training engagement, skill development, adolescent athletes, basketball

## Abstract

**Objective:**

This study aimed to examine the predictive effect of coach–athlete relationship (CAR) quality on training engagement (TE) and shooting skill improvement (SI) among adolescent basketball players, as well as the potential mediating role of TE in this relationship.

**Methods:**

A total of 128 basketball players aged 16–18 years (including 83 males) were recruited. The Coach–Athlete Relationship Questionnaire (CART-Q) was used to assess CAR, the Task and Ego Orientation in Sport Questionnaire (TEOSQ) measured TE, and a 100-shot stationary shooting test was used to evaluate SI at both pre- and post-season. Statistical analyses included Pearson correlation, hierarchical regression, and structural equation modeling (SEM). The significance level was set at *α* = 0.05.

**Results:**

CAR was significantly positively correlated with both TE (r = 0.52, *p* < 0.001) and SI (r = 0.38, *p* < 0.001). After controlling for gender and competition experience, CAR remained a significant predictor of TE (*β* = 0.48, *p* < 0.001) and SI (*β* = 0.31, *p* = 0.002). TE partially mediated the relationship between CAR and SI (*β* = 0.14, 95% CI [0.06, 0.24]), accounting for 46% of the total effect. The SEM showed a good model fit (χ^2^/df = 1.86, CFI = 0.95, RMSEA = 0.072).

**Conclusion:**

A high-quality coach–athlete relationship not only directly enhances training engagement in adolescent basketball players but also indirectly facilitates shooting skill improvement over the course of a season by increasing training engagement.

## 1 Introduction

The quality of the coach–athlete relationship (CAR) serves as a key indicator of the emotional bond and collaborative engagement between coaches and athletes. It has long been recognized as a critical factor in enhancing training effectiveness and supporting athletes’ overall development (Jowett and Slade, 2021). The “3Cs + 1″ model proposed by Jowett et al. (2012) conceptualizes CAR quality across four dimensions: closeness, commitment, complementarity, and co-orientation. A high-quality CAR is typically characterized by mutual trust, respect, and understanding, shared goal setting and aligned behavior, and reciprocal support (Davis et al., 2018; LemanaIi et al., 2023). Extensive research has demonstrated that a positive CAR significantly enhances athletes’ training engagement, sport satisfaction, self-esteem, and competitive performance (Jiahao and Jing, 2024; Papaioannou and Hackfort, 2014). For example, studies have shown that effective communication and mutual respect between coach and athlete are associated with greater athlete satisfaction and improved competitive outcomes (Liu et al., 2025). Moreover, a strong coach–athlete relationship fosters increased athlete involvement and confidence, which in turn contribute to enhanced training outcomes and performance gains (Phillips et al., 2023). Accordingly, CAR quality is increasingly recognized as a core indicator of coaching effectiveness and a fundamental determinant of athlete development quality.

Training engagement (TE) is a key psychological construct that reflects the cognitive, emotional, and behavioral energy athletes invest in their daily training. It encompasses the degree of attentional focus, emotional involvement, and physical effort that athletes dedicate to the training process (Liu et al., 2024). Conceptually aligned with the notion of “work engagement,” TE is widely recognized as the positive counterpart to athlete burnout and includes core components such as vigor, dedication, and absorption (Raimundi et al., 2024). Lonsdale et al. (2007) defined athlete engagement as a sustained, positive cognitive–emotional experience marked by self-confidence, a willingness to invest time and effort in meaningful goals (dedication), high energy and enthusiasm (vigor), and a passionate, optimistic attitude (enthusiasm). Supporting this view, Weiss and Halupnik (2013) found that athletes with higher levels of training engagement tend to exhibit stronger training adherence and better competitive performance. A variety of factors influence training engagement. While most prior research has focused on individual-level predictors—such as achievement motivation, gratitude, self-efficacy, and coping styles (Rechenmacher et al., 2022)—relatively less attention has been paid to interpersonal and contextual factors. In particular, the coach–athlete relationship (CAR) within training environments is an important but underexplored influence on TE (Lee et al., 2023). As a salient form of external social support, CAR can significantly enhance athletes’ engagement by satisfying basic psychological needs and promoting intrinsic motivation (Jiahao and Jing, 2024). Recent studies have begun to explore the underlying mechanisms of this effect. For instance, Gu et al. (2023) reported that in Chinese team sport settings, a high-quality CAR not only directly improved athlete engagement but also exerted an indirect effect through the experience of thriving—a psychological state characterized by vitality and personal growth. These findings underscore the importance of CAR as a contextual catalyst for fostering young athletes’ enthusiasm, attentional focus, and perseverance in the training process.

Beyond its influence on subjective training engagement, the quality of the coach–athlete relationship (CAR) may also contribute to objective skill development through multiple mechanisms. A high-quality CAR can enhance athletes’ attentional focus and training effort, thereby facilitating more effective learning outcomes (Jowett and Cockerill, 2003). Moreover, positive interpersonal dynamics allow coaches to deliver more targeted technical instruction and personalized feedback, increasing the efficiency of skill acquisition (Jowett and Lavallee, 2007; Mageau and Vallerand, 2003). Evidence from Western contexts indicates that supportive coaching behaviors are significantly associated with performance improvements. For instance, democratic and autonomy-supportive coaching styles have been shown to promote skill development, whereas authoritarian or controlling styles tend to impede athletic progress (Davis et al., 2018; Fan et al., 2023). These findings suggest that CAR quality functions as a motivational mechanism, fostering a positive training climate that facilitates skill advancement. However, the majority of empirical studies on CAR have been conducted in Western cultural contexts, where egalitarian and collaborative coach–athlete dynamics are emphasized (Babbitt, 2019; Su and Zhao, 2023). In contrast, the Chinese coach–athlete relationship often reflects a hierarchical mentor–disciple (Shifu–Tudi) model, which places strong emphasis on authority, discipline, and obedience. Qualitative studies have shown that elite Chinese athletes commonly view their coaches as paternalistic mentors who play a central role not only in training but also in their personal and psychological development (Wang and Dong, 2018; Ye et al., 2016). Conversely, athletes in Western contexts are more likely to perceive their coaches as equal collaborators (Landman et al., 2024). These cultural distinctions suggest that the mechanisms through which CAR influences athlete motivation and performance may vary significantly across sociocultural settings (Dong et al., 2024). In collectivist cultures, where interpersonal harmony and respect for authority are highly valued, both support and pressure from coaches may carry greater psychological weight (Sasaba et al., 2017). Within such frameworks, a high-quality CAR may exert a stronger facilitative effect by fostering trust, reducing interpersonal tension, and enabling athletes to fully concentrate on training. Furthermore, guidance delivered within a relationship grounded in deep personal trust is more likely to be internalized by athletes, thereby optimizing skill development outcomes (Wu et al., 2017).

Given the preceding analysis, it is essential to examine the predictive role of coach–athlete relationship (CAR) quality in shaping training engagement and skill development among youth athletes within the Chinese sociocultural context. Such an investigation would enrich the theoretical understanding of cross-cultural dynamics in sport psychology and offer empirical support for evidence-based coaching and athlete development practices in China. Accordingly, the present study employed a longitudinal design to track Chinese adolescent basketball players over the course of a competitive season. It systematically examined the influence of CAR quality on athletes’ training engagement and improvements in shooting skill performance, while also exploring the mediating role of training engagement in this relationship. It was hypothesized that a high-quality coach–athlete relationship would positively predict training engagement, which in turn would lead to significant improvements in basketball skill performance among youth athletes.

## 2 Methods

This study was conducted in full compliance with the ethical principles outlined in the Declaration of Helsinki and was approved by the Ethics Committee of Beijing Sport University (Approval No.: 2024241H). All participants provided written informed consent after being thoroughly informed of the study’s objectives, procedures, and potential risks.

### 2.1 Participants

Sample size estimation was performed using G*Power 3.1.9.2. Based on a medium effect size (f^2^ = 0.15), a significance level of *α* = 0.05, and a statistical power of 1 – *β* = 0.80, hierarchical regression analysis indicated a minimum required sample size of 107 participants. In practice, 132 basketball athletes aged 16–18 were recruited from three elite youth teams in Beijing. During the study, four participants were excluded due to injury or more than 10% absence from training. The final sample included 128 athletes (83 males; mean age = 17.2 ± 0.7 years). Inclusion criteria were as follows:(1) right-handed; (2) normal or corrected-to-normal vision, with no color blindness; (3) no history of neurological or psychiatric disorders; (4) at least two years of systematic basketball training and no upper-limb injury history; (5) written informed consent obtained prior to participation.

### 2.2 Research design

This study adopted a longitudinal design spanning the duration of a competitive basketball season. Measurements of the coach–athlete relationship (CAR), training engagement (TE), and shooting improvement (SI) were collected at two time points: prior to the start of the season (T1, March) and following its conclusion (T2, September). The primary aim was to examine the predictive effects of CAR on both TE and SI, as well as to explore the potential mediating role of TE in the relationship between CAR and shooting performance over time.

### 2.3 Assessment instruments

**Figure 1:**
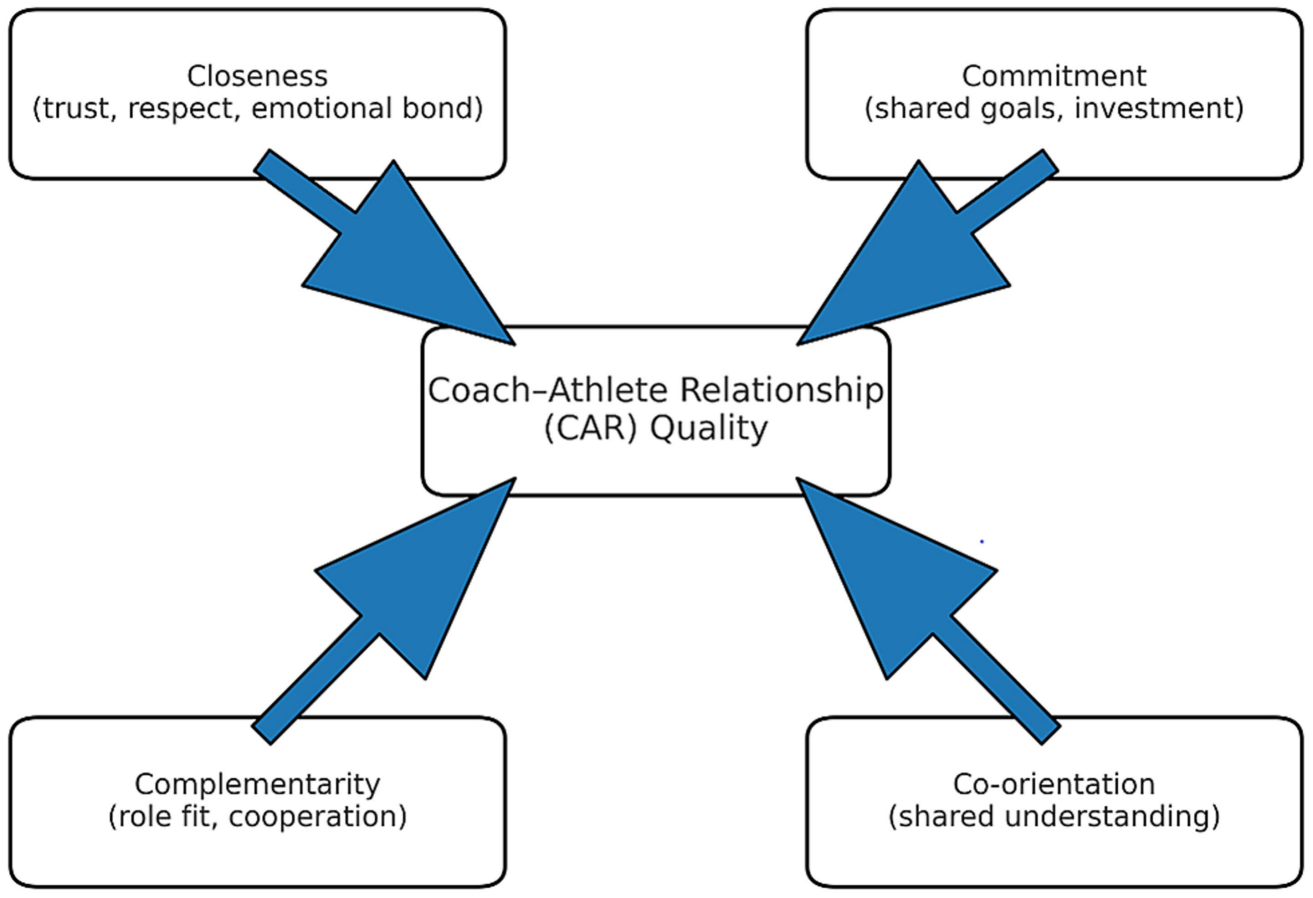
The “3Cs + 1” model of coach–athlete relationship (CAR) quality (Jowett et al., 2012).

The quality of the coach–athlete relationship was assessed using the Chinese version of the Coach–Athlete Relationship Questionnaire (CART-Q) (Zhong and Wang, 2007). This study was grounded in the “3Cs ± 1” model proposed by Jowett et al. (2012) (Figure 1), which conceptualizes the coach–athlete relationship (CAR) across four interrelated dimensions: closeness, commitment, complementarity, and co-orientation. Closeness refers to the emotional bond and level of trust between coach and athlete, manifested through mutual respect, liking, and emotional support. Commitment denotes the mutual investment of effort and steadfast willingness to maintain a long-term cooperative relationship, encompassing dedication to shared goals and a strong sense of responsibility. Complementarity reflects the coordination and harmonious interaction between coach and athlete in their respective roles and behaviors, as evidenced by effective collaboration during training. Co-orientation captures the extent to which both parties share a common understanding of the nature and objectives of their relationship—that is, the mutual perception of closeness, commitment, and complementarity. Operationally, CAR quality was assessed from the athletes’ perspective using the standardized Coach–Athlete Relationship Questionnaire (CART-Q). This self-report instrument includes multiple items representing the model’s dimensions, such as “My coach and I have a strong mutual trust” (closeness), “I hope to work with my coach for a long time” (commitment), and “My coach and I cooperate well during training” (complementarity). Responses are rated on a Likert-type scale, with higher scores indicating greater perceived relationship quality. By aggregating scores across dimensions, the CART-Q provides an integrated, quantitative measure of CAR quality that systematically operationalizes the core constructs of the “3Cs ± 1″ model. The scale comprises 11 items rated on a 7-point Likert scale, with higher scores reflecting stronger perceived relationship quality. In the current study, the CART-Q demonstrated excellent internal consistency (Cronbach’s *α* = 0.92), and confirmatory factor analysis indicated satisfactory construct validity (χ^2^/df = 2.01, CFI = 0.96) (Pinho et al., 2024; Walter et al., 2025; Yang and Jowett, 2012). Training Engagement (TE): Training engagement was measured using the revised version of the Training Engagement in Sport Questionnaire (TEOSQ), which consists of 18 items rated on a 5-point Likert scale. The instrument assesses three core dimensions of engagement—cognitive, emotional, and behavioral (Cid et al., 2010; Morales-Sánchez et al., 2022). All subscales showed high internal consistency, with Cronbach’s α values ranging from 0.90 to 0.93. Shooting Improvement (SI): Shooting performance was evaluated using a 100-shot stationary shooting test (Ma and Monsma, 2016; Wang et al., 2010). Participants attempted 20 shots from each of five standardized court positions, and the number of successful hits was recorded. Performance was independently assessed by two trained raters, yielding excellent inter-rater reliability (intraclass correlation coefficient [ICC] = 0.92). Shooting improvement was calculated as the percentage change in shooting accuracy from pre- to post-season, using the following formula:


$$\mathrm{SI}=\frac{\text{Post}-\mathrm{Pr}e}{\mathrm{Pr}e}\times 100$$

### 2.4 Testing procedure

This study involved two assessment time points. At T1 (baseline), participants completed the Coach–Athlete Relationship (CAR) and Training Engagement (TE) questionnaires in a centralized setting using the Wenjuanxing online survey platform. This was immediately followed by the 100-shot stationary shooting pre-test. During the intervention period, each team maintained its standard training regimen. To avoid contamination or researcher bias, the research team refrained from providing any technical coaching and was solely responsible for monitoring athlete attendance, which averaged 94.6% across participants. At T2 (post-season), the same questionnaires were re-administered, and the post-test shooting assessment was conducted under conditions identical to the pre-test. All evaluations were performed by the same research team using the same equipment and facilities to ensure consistency and comparability across time points. To ensure data integrity, questionnaire responses were reviewed on-site by trained research assistants, who addressed any missing or invalid entries in real time. The shooting assessments were fully recorded using two fixed-angle video cameras, and scoring was performed via asynchronous coding by two independent raters to enhance objectivity and inter-rater reliability.

### 2.5 Statistical analysis

Prior to analysis, data preprocessing was conducted. The proportion of missing values across all variables was below 3%, and missing data were imputed using the Expectation–Maximization (EM) algorithm. The Shapiro–Wilk test indicated acceptable normality for all variables, with skewness and kurtosis values below 1. Outliers, identified as standardized Pearson residuals exceeding |3.29| (n = 2), were minorized in accordance with established guidelines. Descriptive statistics and two-tailed Pearson correlation coefficients were computed for all primary variables. The significance threshold was set at *α* = 0.05. To assess the predictive effects of Coach–Athlete Relationship (CAR) quality on Training Engagement (TE) and Shooting Improvement (SI), hierarchical regression analyses were conducted using SPSS 28. Gender and competition experience were entered as control variables in Step 1, while CAR was added in Step 2 to assess its incremental explanatory power. To further examine the hypothesized mediation model, structural equation modeling (SEM) was performed using Muplus 8.7. The indirect effect of TE on the relationship between CAR and SI was tested using a bias-corrected nonparametric bootstrap procedure with 5,000 resamples. Model fit was evaluated using the following criteria: χ^2^/df < 3, CFI and TLI > 0.90, RMSEA < 0.08, and SRMR < 0.08. Effect sizes for the regression analyses were reported using Cohen’s f^2^. For mediation analyses, 95% bootstrap confidence intervals (CIs) were provided for indirect effects. Where applicable, significance levels were adjusted using the Holm–Bonferroni method to account for multiple comparisons.

## 3 Results

### 3.1 Descriptive statistics, distribution characteristics, and normality tests of study variables

As presented in Table 1, descriptive statistics were calculated for Coach–Athlete Relationship (CAR), Training Engagement (TE), Shooting Improvement (SI), and shooting accuracy at the beginning (T1) and end (T2) of the season. The analyses included means ± standard deviations, distributional characteristics, and normality checks controlling for gender. All variables demonstrated acceptable distributional properties, with absolute skewness and kurtosis values below 1.0. Results of the Shapiro–Wilk tests were non-significant (all *p* > 0.05), indicating that the assumption of normality was met. Independent-samples t-tests showed no significant gender differences across any of the key variables (all t < 1.75, *p* > 0.08), suggesting that gender did not significantly influence the measured outcomes in this sample.

**Table 1:** Descriptive statistics of key variables.

Variable	Time point	M ± SD	Skewness	Kurtosis	Normality
Coach–athlete relationship (CAR)	T1	5.82 ± 0.71	−0.32	−0.57	0.26
Training engagement (TE)	T1	3.78 ± 0.55	−0.21	−0.48	0.18
Shooting accuracy (shots/100)	T1	56.1 ± 7.4	0.06	−0.41	0.72
Shooting accuracy (shots/100)	T2	64.4 ± 6.8	−0.19	−0.38	0.31
Shooting improvement rate (SI, %)	—	14.8 ± 7.3	0.27	−0.71	0.24

### 3.2 Correlational analysis among coach–athlete relationship, training engagement, and shooting improvement

**Figure 2:**
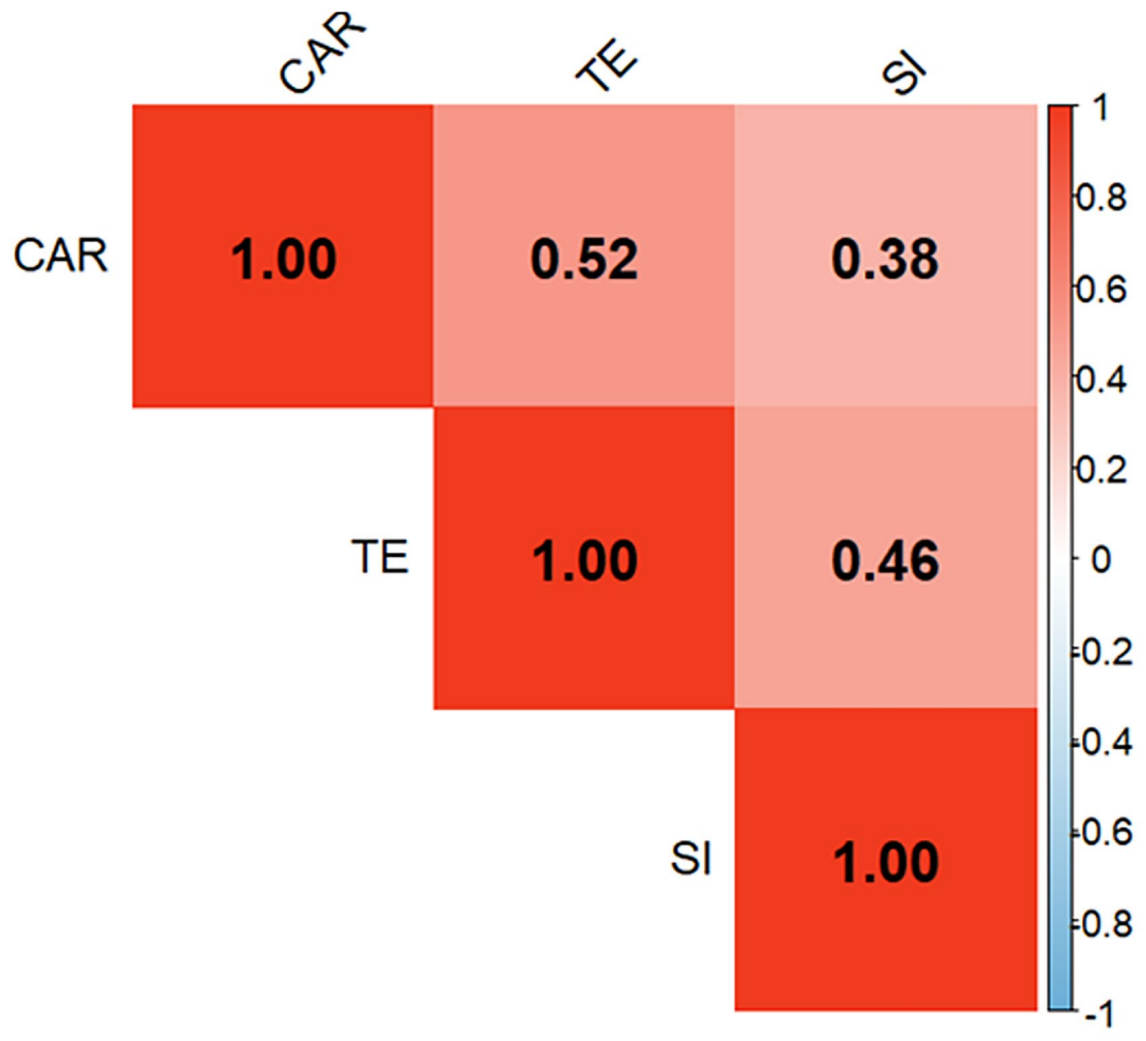
Pearson correlation matrix of key variables.

As illustrated in Figure 2, the Coach–Athlete Relationship (CAR), Training Engagement (TE), and Shooting Improvement (SI) were all significantly and positively correlated, with moderate to strong effect sizes. CAR showed the strongest association with TE (r = 0.52, *p* < 0.001), followed by the correlation between TE and SI (r = 0.46, *p* < 0.001), and between CAR and SI (r = 0.38, *p* < 0.001).

### 3.3 Hierarchical regression analyses: incremental predictive effects of CAR on TE and SI after controlling for covariates

**Figure 3:**
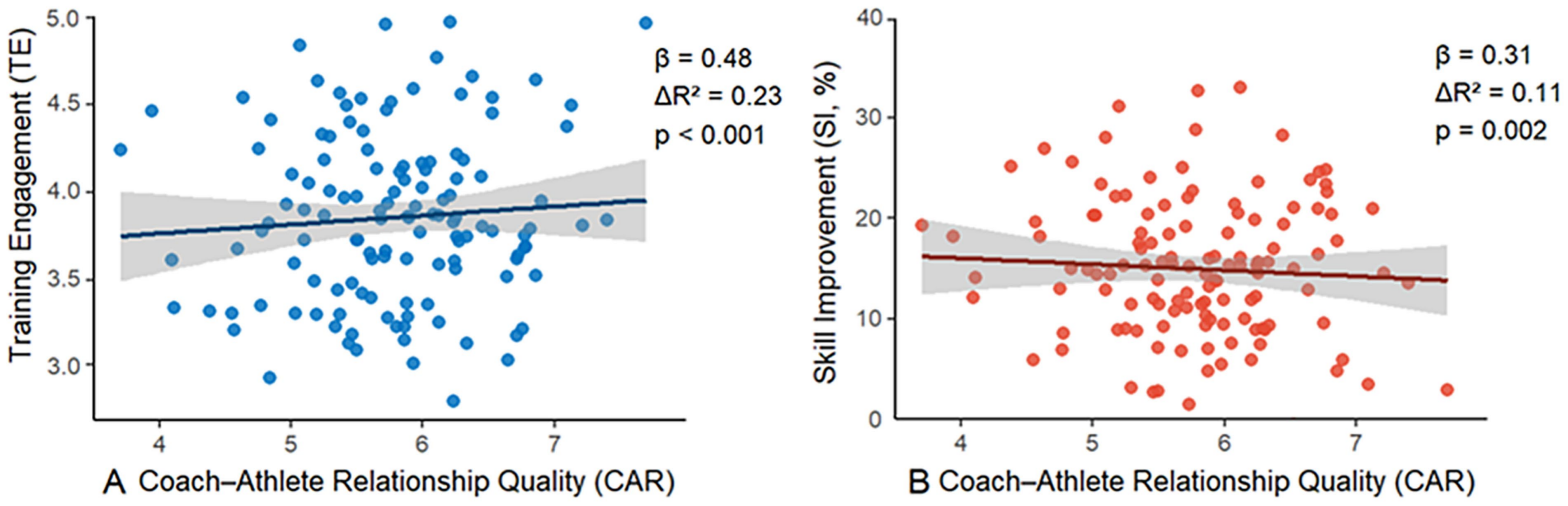
Results of hierarchical regression analyses.

As illustrated in Figure 3, after controlling for gender and competitive experience, the Coach–Athlete Relationship (CAR) demonstrated significant incremental predictive effects on both Training Engagement (TE) and Shooting Improvement (SI). For the TE model, CAR accounted for an additional 23% of the variance (ΔR^2^ = 0.23), corresponding to a medium effect size (Cohen’s f^2^ = 0.30). The standardized regression coefficient was *β* = 0.48 (*p* < 0.001), indicating that CAR was a strong positive predictor of TE. In the model predicting SI, the additional explained variance was 11% (ΔR^2^ = 0.11), reflecting a small-to-medium effect size (f^2^ = 0.12). CAR also emerged as a significant positive predictor of SI (*β* = 0.31, *p* = 0.002).

### 3.4 Structural equation modeling of the mediating role of training engagement between CAR and shooting improvement

**Figure 4:**
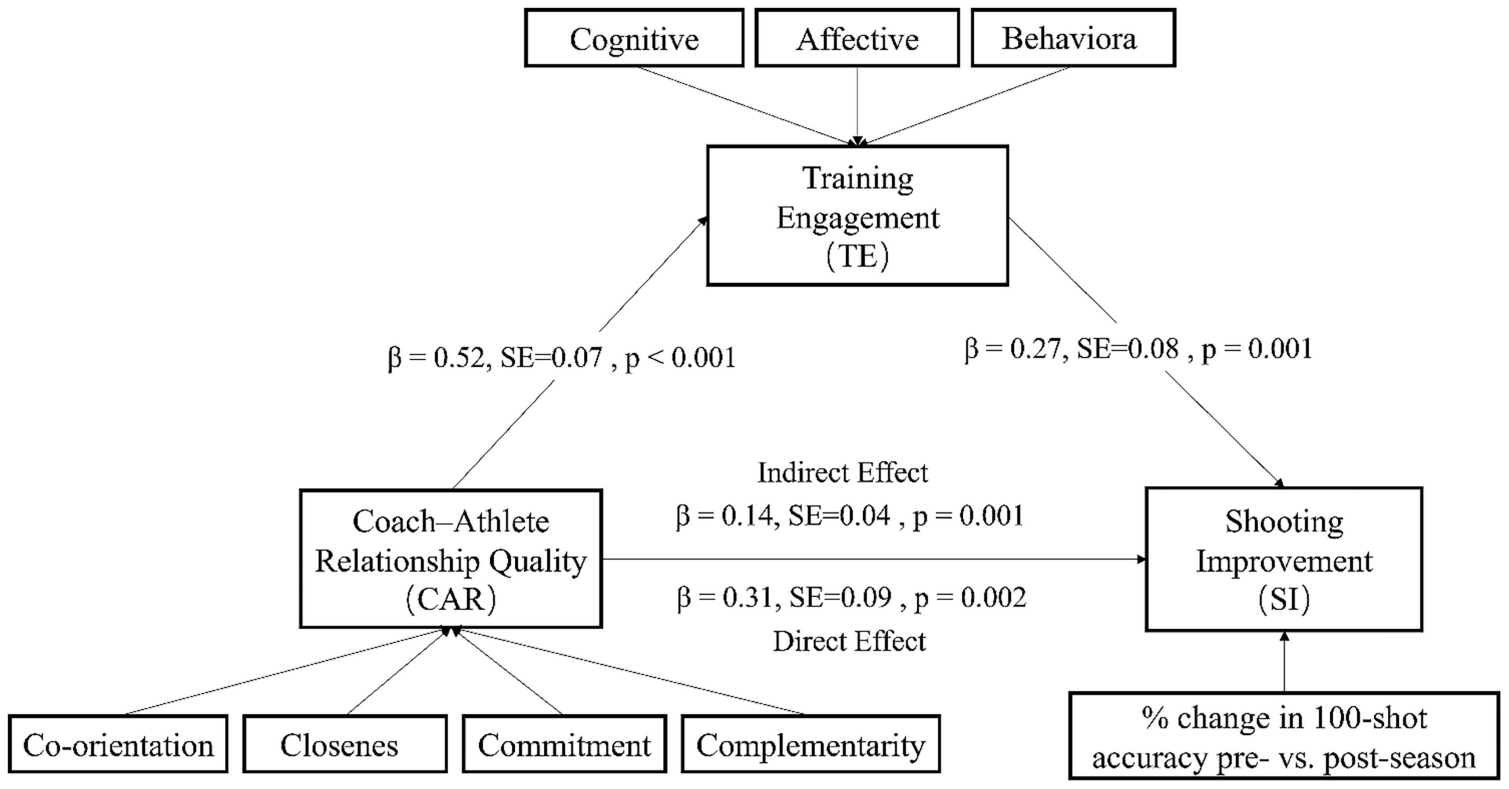
SEM path coefficients and mediation effects.

As shown in Figure 4, the hypothesized structural model specified a sequential pathway in which the coach–athlete relationship (CAR) predicted training engagement (TE), which in turn predicted shooting improvement (SI) (CAR → TE → SI). The results were as follows: (1) the standardized path coefficient from CAR to TE was *β* = 0.52 (*p* ≤ 0.001), indicating that higher-quality CAR significantly and positively predicted athletes’ training engagement; (2) the standardized path coefficient from TE to SI was β = 0.27 (*p* = 0.001), suggesting that greater training engagement was associated with larger gains in shooting accuracy; and (3) when the direct pathway was included, CAR also significantly predicted SI (*β* = 0.31, *p* = 0.002). The model demonstrated good fit to the data (χ^2^/df = 1.86, CFI = 0.95, RMSEA = 0.072). Mediation analysis revealed that the indirect effect of CAR on SI through TE was *β* = 0.14, 95% CI [0.06, 0.24], *p* = 0.001, accounting for approximately 31% of the total effect. This indicates that TE served as a partial mediator: higher-quality CAR not only directly improved shooting performance but also indirectly enhanced performance by increasing training engagement.

### 3.5 Analysis of Shooting Accuracy Improvement, Effect Sizes, and CAR Quartile Group Comparisons

**Figure 5:**
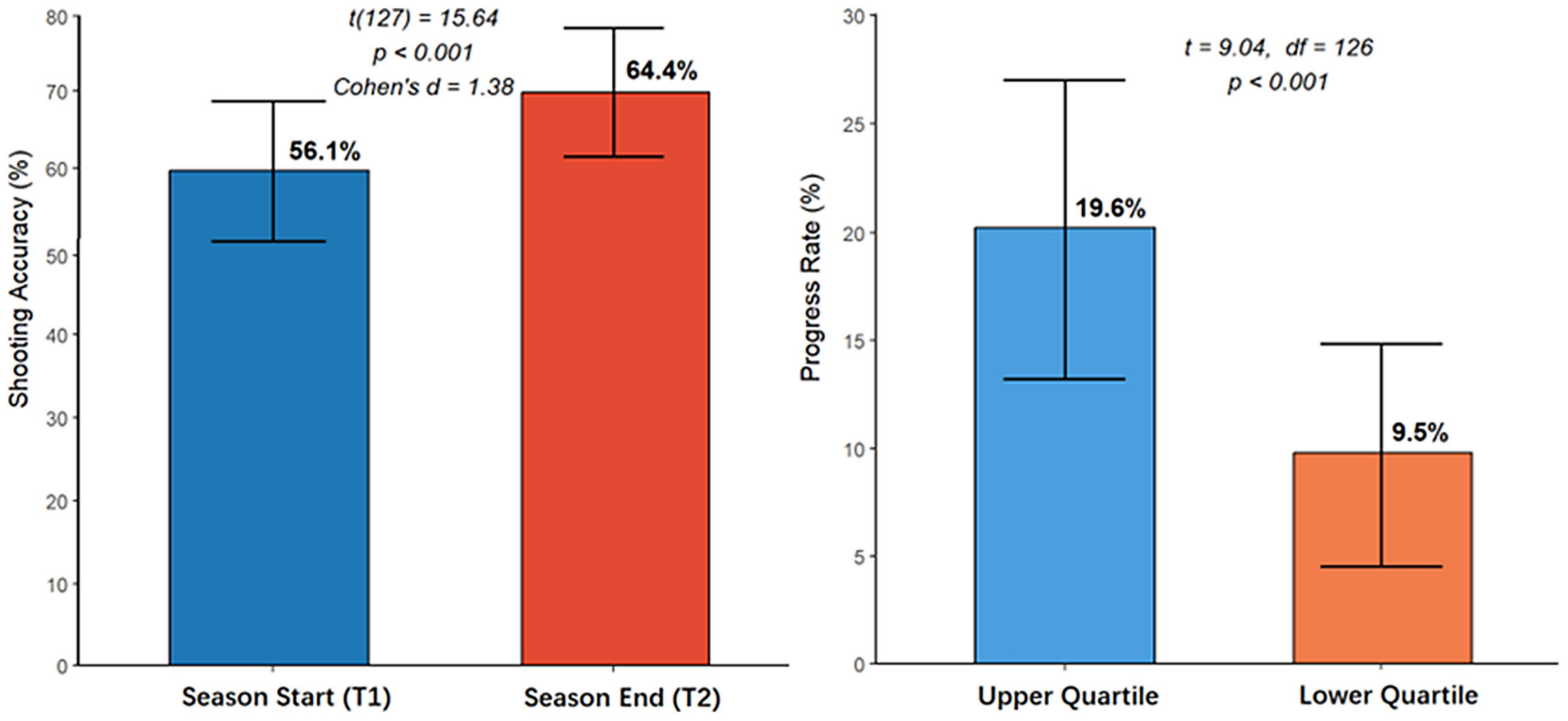
Season-to-season shooting improvement and influence of coach–athlete relationship quality.

As illustrated in Figure 5 Shooting accuracy increased from 56.1 to 64.4% over the course of the season, corresponding to a mean percentage improvement of 14.8%. A paired-samples t-test confirmed that this improvement was statistically significant, t(127) = 15.64, *p* < 0.001, with a large effect size (Cohen’s d = 1.38). Quartile analysis further revealed that athletes in the highest quartile of Coach–Athlete Relationship (CAR) quality showed significantly greater improvement than those in the lowest quartile (19.6% ± 6.8% vs. 9.5% ± 5.1%, t = 9.04, *p* < 0.001), providing additional support for the moderating effect of CAR on training-related performance gains.

## 4 Discussion

The findings of this study reveal significant positive associations among coach–athlete relationship quality (CAR), training engagement (TE), and shooting improvement (SI), with CAR correlating strongly with TE (r = 0.52, *p* < 0.001) and moderately with SI (r = 0.38, *p* < 0.001). Hierarchical regression analysis, controlling for gender and competitive experience, confirmed that CAR was a significant predictor of both TE (*β* = 0.48, *p* < 0.001) and SI (*β* = 0.31, *p* = 0.002), underscoring its critical role as a facilitating factor in athlete development. Mediation analysis further demonstrated that TE partially mediated the relationship between CAR and SI (*β* = 0.14, 95% CI [0.06, 0.24]), accounting for approximately 46% of the total effect. The structural equation model exhibited acceptable fit indices (χ^2^/df = 1.86, CFI = 0.95, RMSEA = 0.072), providing empirical support for the hypothesized mechanism in which CAR indirectly enhances skill performance by promoting greater training engagement.

Extensive prior research has demonstrated that high-quality coach–athlete relationships (CAR) play a crucial role in promoting athletes’ training engagement (TE) and performance outcomes (Davis et al., 2019; Gerber et al., 2024; Liu et al., 2025). In the present study involving Chinese adolescent basketball players, CAR was moderately correlated with TE (r = 0.52) and weakly to moderately correlated with shooting improvement (SI; r = 0.38). Even after controlling for gender and competition experience, CAR remained a significant predictor of both TE (*β* = 0.48, *p* < 0.001) and SI (*β* = 0.31, *p* = 0.002). Mediation analysis further revealed that TE partially mediated the relationship between CAR and SI, accounting for approximately 46% of the total effect. These findings are consistent with existing empirical evidence. For example, Gu et al. (2023) found that, in Chinese team-sport athletes, CAR positively influenced TE both directly and indirectly through the promotion of thriving—a psychological state characterized by vitality and perceived personal growth. Comparable mechanisms have also been observed in Western contexts. Drawing on Self-Determination Theory, Mageau and Vallerand (2003) emphasized that autonomy-supportive coaching behaviors satisfy athletes’ basic psychological needs for autonomy, competence, and relatedness, thereby enhancing intrinsic motivation. Likewise, Jowett et al. (2012) demonstrated that the core dimensions of CAR—closeness, commitment, and complementarity—are strongly associated with higher levels of training engagement. While the beneficial effects of CAR on TE appear to be robust across studies, the underlying mechanisms may be moderated by cultural norms and theoretical perspectives. The current study thus extends this line of research by situating the CAR–TE–SI pathway within the Chinese sociocultural context, offering new insight into how interpersonal dynamics shape athletic development in collectivist cultures.

In Western contexts, research on the coach–athlete relationship (CAR) has frequently drawn upon Self-Determination Theory, which emphasizes that the fulfillment of athletes’ basic psychological needs—autonomy, competence, and relatedness—fosters intrinsic motivation and, in turn, enhances training engagement (Dong et al., 2024). For instance, Longakit et al. (2024) identified a mediating role of CAR in the relationship between motivation and engagement, suggesting that a supportive coach–athlete bond amplifies the positive effects of both intrinsic and extrinsic motivation on behavioral involvement. Emotional–motivational pathways have also been highlighted: Lemana Ii et al. (2024) found that emotion regulation mediates the association between CAR and athletic performance, while Zhao and Jowett (2023) emphasized that psychological need satisfaction enhances the quality of training participation. In contrast, Chinese research has underscored the influence of traditional “master–apprentice” (Shifu–Tudi) dynamics and collectivist cultural values in shaping CAR. Within this context, coaches are not only viewed as technical instructors but also as authoritative mentors who fulfill educational, managerial, and emotional roles (Guo and Yang, 2021; Wang and Liu, 2024). Interestingly, diverging from Western findings that often critique authoritarian leadership for undermining relational quality, Liu et al. (2025) reported that both democratic and authoritarian coaching styles were positively associated with CAR quality and athletic outcomes among Chinese adolescents. This suggests that in cultural settings where discipline, hierarchy, and accountability are highly valued, authoritative behaviors may be interpreted as forms of care and responsible guidance rather than coercion. Moreover, given the cultural emphasis on interpersonal harmony and collective achievement, Chinese athletes tend to prioritize relational stability and team cohesion, making them especially responsive to their coaches’ support, expectations, and feedback(Xin et al., 2024; Ye, 2016). Within such a sociocultural framework, high-quality CAR may not only reduce interpersonal conflict and psychological pressure—thereby fostering a focused, low-stress training environment—but also promote deeper internalization of training content through personalized instruction. These culturally embedded mechanisms ultimately enhance athletes’ behavioral engagement and underscore the importance of aligning relational strategies with sociocultural values and athlete expectations.

This study investigated training engagement (TE) as a key mediating variable in the relationship between coach–athlete relationship (CAR) quality and shooting improvement (SI), emphasizing athletes’ cognitive, emotional, and behavioral investment in the training process. Prior research has proposed several alternative or supplementary mediators—such as thriving, motivational regulation, and hope—all situated along the pathway from interpersonal social contexts (e.g., CAR) to athletic outcomes. These constructs highlight the motivational and psychological mechanisms through which CAR supports and activates athletes’ performance potential. For example, Gu et al. (2023) found that CAR enhances TE by promoting athletes’ thriving, defined as a psychological state marked by vitality and a sense of progress. This, in turn, facilitates improved athletic performance. Within the framework of Self-Determination Theory, such a pathway suggests that CAR supports athletes’ basic psychological needs (i.e., autonomy, competence, and relatedness), thereby energizing sustained engagement. In a similar vein, Tian et al. (2019) demonstrated that CAR shapes athletes’ motivational regulation by encouraging the internalization of external demands into self-congruent goals—complementing TE’s role in channeling energy into training tasks. Emerging research within positive psychology has also underscored the role of hope as a mediator. Xin et al. (2024) found that CAR, via a gratitude-enhancing mechanism, elevates athletes’ levels of hope—defined as an individual’s perceived capability to generate goal-directed pathways and sustain motivation—thus reducing the risk of training burnout. Although the present study did not directly examine thriving, motivational regulation, or hope, these constructs are conceptually consistent with TE as indicators of positive engagement and psychological activation, and warrant consideration in future investigations (Bektiningtyas and Undarwati, 2023). Importantly, each mediator provides a distinct theoretical lens: (1)Training Engagement captures observable effort and behavioral investment in structured training routines; (2)Thriving reflects athletes’ subjective sense of vitality and growth;(3) Motivational Regulation explains how motivation is sourced and internalized;(4) Hope emphasizes cognitive expectation and emotional agency toward goal pursuit (Wu et al., 2023).

Cultural differences between Chinese and Western societies fundamentally shape the psychological functions of the coach–athlete relationship (CAR) and the mechanisms through which it influences athletes’ motivation and behavior (Zhao and Jowett, 2023). In Western contexts, where individual autonomy, equality, and self-expression are highly valued, coaches are typically regarded as facilitators or collaborators. This coaching style supports athletes’ autonomy and decision-making, aligning with the core needs outlined by Self-Determination Theory—namely, autonomy, competence, and relatedness—which collectively promote intrinsic motivation and sustained engagement in training (Babbitt, 2019; Mageau and Vallerand, 2007; Su and Zhao, 2023). In contrast, the Chinese cultural framework—shaped by Confucian “master–apprentice” traditions and collectivist values—tends to portray coaches as paternalistic mentors who simultaneously fulfill instructional, administrative, and emotional caregiving roles (Wang and Liu, 2024; Ye et al., 2016) In this context, CAR is closely associated with trust, loyalty, and hierarchical respect, placing greater emphasis on discipline and order (Hartanto et al., 2025). Athletes often view relational harmony as a critical interpersonal asset and attach significant psychological weight to their coaches’ emotional support and behavioral expectations. This dynamic promotes a low-conflict, high-focus training atmosphere that facilitates attentional control and long-term engagement (Jowett et al., 2023). These cultural distinctions also influence how athletes interpret coaching behaviors. Whereas authoritarian leadership is often perceived as undermining autonomy and hindering performance in Western societies (Davis et al., 2018), studies conducted in China suggest that disciplined and high-demand coaching styles may—under culturally congruent conditions—positively predict both training commitment and performance outcomes (Fan et al., 2023). One possible explanation is the mechanism of “benevolent authority internalization”: athletes may interpret strict discipline not as coercive control but as an expression of professional competence and genuine care. In collectivist contexts, such interpretations may buffer the potential negative effects of authoritative behaviors and instead enhance motivational pathways. In sum, these findings underscore the necessity of cultural sensitivity in both theoretical modeling and practical application of CAR. Cross-cultural variations must be taken into account when adapting coaching styles or designing athlete development frameworks, especially in increasingly globalized sports environments.

Drawing on empirical data from Chinese adolescent basketball athletes, this study found that high-quality coach–athlete relationships (CAR) exert significant positive effects on both training engagement (TE) and shooting improvement (SI), consistent with findings reported in both domestic and international literature. However, the psychological mechanisms through which CAR exerts its influence appear to differ across cultural contexts. In the Chinese setting, CAR is often framed within authoritative, mentor-based (“master–apprentice”) dynamics that are thought to mitigate interpersonal conflict and psychological stress. In contrast, Western research has predominantly emphasized autonomy-supportive coaching practices that fulfill basic psychological needs and promote intrinsic motivation (Jowett and Carpenter, 2015). The TE-based mediation model proposed in this study conceptually overlaps with other psychological constructs—such as thriving, motivational regulation, and hope—that have been identified as mediators linking social-contextual factors (e.g., CAR) with athletic performance. While these constructs share a common emphasis on the role of psychological resources, each highlights a distinct dimension of athlete functioning: TE focuses on observable behavioral investment in training; thriving captures athletes’ subjective vitality and growth experiences; motivational regulation addresses the internalization and transformation of motivation types; and hope reflects cognitive and emotional orientations toward goal pursuit. From a cross-cultural perspective, the psychological functions of CAR extend beyond the universal needs outlined in Self-Determination Theory and are shaped by sociocultural norms, values, and interpersonal expectations. In collectivist cultures like China, CAR may derive its efficacy not solely from autonomy support but from relational harmony, hierarchical trust, and internalized authority structures. Accordingly, future research should further explore the culturally situated mechanisms through which CAR influences athletic development, using comparative and mixed-method approaches to uncover context-specific dynamics. Practically, these insights may inform the development of culturally responsive coaching strategies that not only enhance performance outcomes but also promote the holistic growth of adolescent athletes within diverse sporting environments.

## 5 Limitations and future directions

Despite the strengths of this study—including its longitudinal design and the inclusion of relevant covariates—several limitations should be acknowledged. First, the sample was limited to three adolescent basketball teams based in Beijing, which may constrain the generalizability of the findings due to restricted sample size and regional scope. Second, both the Coach–Athlete Relationship (CAR) and Training Engagement (TE) were measured through self-report questionnaires, raising the possibility of common method bias and limiting the strength of causal inferences. Third, the assessment of skill development focused solely on shooting performance, without consideration of other technical skills or competitive outcomes, thus narrowing the scope of performance evaluation. To enhance external validity and interpretive depth, future research should adopt broader and more diverse sampling strategies across different regions, sports disciplines, and competitive levels. Incorporating multi-informant data sources—such as coach evaluations, behavioral coding, and physiological or neurocognitive markers—would provide a more comprehensive and objective assessment framework. Additionally, experimental or intervention-based designs are needed to clarify the causal pathways linking CAR to training engagement and performance outcomes. Future studies should also consider examining potential moderating variables, including cultural context, gender, and developmental stage, to better understand how these factors shape the influence of CAR. Such efforts would contribute to the development of a more culturally responsive and empirically grounded coaching framework aimed at promoting both performance and holistic development among adolescent athletes. Future research should adopt larger, more diverse samples and integrate multi-source data—including coach ratings, behavioral observations, and physiological indicators—to explore additional underlying mechanisms. Such efforts will deepen the theoretical understanding of youth athletic development and offer a stronger evidence base for designing effective, culturally informed coaching practices aimed at optimizing training engagement and performance outcomes.

## 6 Conclusion

Longitudinal analysis across the competitive season revealed that CAR significantly predicted TE (*β* = 0.52, *p* ≤ 0.001), and TE, in turn, significantly predicted SI (β = 0.27, *p* = 0.001). CAR also exerted a direct effect on SI (β = 0.31, *p* = 0.002). Mediation analysis indicated that TE partially mediated the CAR–SI relationship, with an indirect effect of β = 0.14, accounting for 31% of the total effect. The model demonstrated satisfactory fit (χ^2^/df = 1.86, CFI = 0.95, RMSEA = 0.072). From an applied perspective, coaches are advised to adopt micro-intervention strategies that strengthen the “3Cs ± 1” dimensions—closeness, commitment, complementarity, and co-orientation—while simultaneously targeting the cognitive, affective, and behavioral facets of TE. Practical approaches include delivering individualized feedback, co-setting and reviewing stage-specific performance goals, clarifying role expectations and implementing coordinated drills, and facilitating information sharing to foster cognitive alignment. Additionally, routine use of the Task and Ego Orientation in Sport Questionnaire (TEOSQ) for monthly monitoring can support a structured “goal–feedback–adjustment” loop, thereby sustaining high levels of training engagement and accelerating shooting skill development in youth athletes.

## Data Availability

The raw data supporting the conclusions of this article will be made available by the authors, without undue reservation.
